# Design of the Multi-Bioactive Graphene-Oxide/Gelatin/Alginate Scaffolds as Dual ECM-Mimetic and Specific Wound Healing Phase-Target Therapeutic Concept for Advanced Wound Healing

**DOI:** 10.3390/pharmaceutics17010089

**Published:** 2025-01-12

**Authors:** Marko Demenj, Martina Žabčić, Marija Vukomanović, Tatjana Ilić-Tomić, Dušan Milivojević, Simonida Tomić, Dubravka Živanović, Marija M. Babić Radić

**Affiliations:** 1 University of Belgrade, University Clinical Center of Serbia, Clinic of Dermatology and Venereology, Pasterova 2, 11000 Belgrade, Serbia; markodemenj@gmail.com (M.D.); dubravka.zivanovic@med.bg.ac.rs (D.Ž.); 2 Advanced Materials Department, Jožef Stefan Institute, Jamova Cesta 39, 1000 Ljubljana, Slovenia; martina.zabcic@ijs.si (M.Ž.); marija.vukomanovic@ijs.si (M.V.); 3 University of Belgrade, Institute of Molecular Genetics and Genetic Engineering, Vojvode Stepe 444a, 11000 Belgrade, Serbia; tatjana.ilic-tomic@imgge.bg.ac.rs (T.I.-T.); dusan.milivojevic@imgge.bg.ac.rs (D.M.); 4 University of Belgrade, Faculty of Technology and Metallurgy, Karnegijeva 4, 11000 Belgrade, Serbia; simonida@tmf.bg.ac.rs

**Keywords:** polymeric scaffolds, gelatin/alinate based scaffolds, ECM-mimetic wound therapy, multi-target wound therapy, allantoin, caffeic acid, graphene oxide nanoparticles

## Abstract

**Objectives:** To develop and evaluate graphene oxide/gelatin/alginate scaffolds for advanced wound therapy capable of mimicking the native extracellular matrix (ECM) and bio-stimulating all specific phases of the wound healing process, from inflammation and proliferation to the remodeling of damaged skin tissue in three dimensions. **Methods:** The scaffolds were engineered as interpenetrating polymeric networks by the crosslinking reaction of gelatin in the presence of alginate and characterized by structural, morphological, mechanical, swelling properties, porosity, adhesion to the skin tissue, wettability, and in vitro simultaneous release of the active agents. Biocompatibility of the scaffolds were evaluated in vitro by MTT test on fibroblasts (MRC5 cells) and in vivo using *Caenorhabditis elegans* assay. **Results:** The scaffolds exhibited a highly porous interconnected morphology with adjustable porosity (93–96%) and mechanical strength (1.10–2.90 MPa), hydrophilic nature with high capacity to absorb physiological fluids, and stable adhesion to the skin tissue. The obtained results of MRC5 cell viability indicate that the scaffolds are safe for biomedical applications. No mortality was detected among the *Caenorhabditis elegans* throughout the incubation period, indicating that the scaffolds are not toxic. The results of in vitro release study of allantoin, quercetin, and caffeic acid confirm the scaffolds’ significant potential for simultaneous release. **Conclusion:** The graphene oxide/gelatin/alginate scaffolds are promising candidates for non-invasive, dual ECM-mimetic, and multi-target wound therapy, offering an innovative strategy to address the complexities of wound healing process.

## 1. Introduction

Wound healing is a complex, three-phase process mediated by multifactorial bioactive stimuli that must be carefully regulated and provided at the appropriate time during the regenerative process [1,2,3]. Any disruption in this process can result in chronic wounds and associated complications such as chronic wound-related amputation or even mortality [4,5]. The key elements of regenerative medicine such as tissue engineering, stem cells, autologous cells, growth factors, and gene therapy have been used to treat wounds [4,6,7]. However, addressing all biological entities of wound environment and the complexity of three-dimensional (3D) skin tissue architecture by a single therapeutic approach has not been achieved for most wounds. Therefore, there is an urgent need to develop innovative wound treatment modalities with multifunctional activity that stimulate cellular processes and health tissue integration in three dimensions through all healing phases, from inflammation and proliferation to the remodeling of the damaged skin tissue.

Recent advances in several fields, such as understanding the biological and physiological phenomena in the wound environment during the wound healing process and developing tissue substrates with multi-bioactive effects on the regenerative process, are providing new opportunities to design advanced 3D multi-target wound therapies that closely mimic wound environment, thereby enhancing the wound healing process. Thus, the transition from two-dimensional to 3D wound healing approaches represents a paradigm shift in wound healing research, with the potential to significantly improve treatment outcomes. In this regard, the development of 3D polymeric scaffolds that mimic the native extracellular matrix (ECM) of skin tissue and feature adjustable bioactivity suitable to stimulate all specific phases in wound healing process could revolutionize wound treatment and save millions of lives [5,8]. Porous 3D polymeric scaffolds act as a healthy ECM substitute, replacing or protecting the native ECM of the skin tissue, rebalancing the wound microenvironment and promoting biological regeneration of damaged tissue in three dimensions [8]. These biomaterials are more than a passive morphological and structural support for cellular processes such as adhesion, migration, and proliferation, they also provide signaling support by improving cell-cell communications and cell-matrix interactions resulting in the activation of different biochemical mediators, growth factors, and cytokines, which are essential in physiology of tissue regeneration process. Beyond ability of 3D polymeric scaffolds to stimulate cellular processes and tissue regeneration by mimicking the native ECM they also possess great potential for loading and controlled/targeted release of various active agents that stimulate specific wound healing phases thereby enhancing scaffolds’ bioactivity necessary for improved wound healing treatment.

Polymeric scaffolds based on natural polymers such as gelatin, silk fibroin, collagen, fibrin, and alginate are extraordinary biomaterials for skin tissue regeneration due to their biocompatibility, hydrophilicity, and unique structure enriched by signal sequences that promote cellular processes [9,10,11]. Being of natural origin, gelatin has excellent biocompatibility and some advantages compared to other natural polymers because of its lower immunogenicity and greater potential for adhesion and proliferation of cells by arginine-glycine-aspartic acid (RGD) sequences in its structure [10,12,13]. From a mechanical point of view, gelatin and alginate have inappropriate mechanical behaviors as scaffolding biomaterials. In order to overcome this advantage various inorganic compounds such as different nanoparticles including gold, silver, titanium dioxide, graphene oxide, zinc oxide, have been used as fillers. Graphene oxide (GO) nanoparticles beyond their ability to enhance physicochemical and mechanical properties of the biomaterials, also improve wound healing process by promoting migration and proliferation of keratinocyte cells [14]. Additionally, graphene oxide nanoparticles possess angiogenic property that stimulates wound healing process [14].

The wound microenvironment substantially influences progression of the regenerative process by supporting various cellular processes. Providing adequate bioactive agents that control the lesion microenvironment and managing all stages of the wound healing process is imperative for efficient wound treatment. The promising strategy to achieve extra-bioactivity of the 3D polymeric scaffolds is their combining by the active agents with high wound healing potential. Here, we presented novel concept of ECM-mimetic and specific wound healing phase-target therapy based on graphene-oxide/gelatin/alginate scaffolds loaded with allantoin, quercetin and caffeic acid. These scaffolds simultaneously mimic the native ECM and stimulate all phases of the wound healing process, including inflammation, proliferation, and remodeling of the damaged skin tissue in three dimensions. The active agents were selected to provide bio-stimulation throughout the entire skin tissue regenerative process. Allantoin modulates inflammatory response, promotes local granulation and proliferation of fibroblasts as well as synthesis of collagen and ECM, while caffeic acid provides myeloperoxidase activity, inhibits peroxidation of lipids and improves re-epithelization by decreasing oxidative stress [15,16,17,18]. Quercetin plays important role in scars and fibrosis prevention during the wound healing process by its strong antifibrotic and antioxidant activity [19]. In this perspective, we presented the innovative wound healing strategy with coupled bio-structural activity of the scaffolds and multi-bioactivity of the active agents into the single entity, providing strong biological assistance across each phase of the skin tissue regenerative process.

The natural-origin (gelatin/alginate) scaffolds reinforced with GO nanoparticles were engineered as the interpenetrating polymeric networks (IPNs) with varying content of polymers, using simple crosslinking reaction of gelatin. Furthermore, the scaffolds were loaded by the bioactive agents with high wound healing potential providing bio-stimulation throughout the entire wound healing process. The effect of the chemical composition of the scaffolds on their properties including chemical, morphological, porosity, capacity to absorb physiological fluids, hydrophilicity, adhesivity to the skin tissue, and the active agents release properties was examined. The biocompatibility and the proliferative effects of the scaffolds were confirmed in vitro by MTT test on fibroblasts (MRC5 cell line) and in vivo using *Caenorhabditis elegans* (*C. elegans*). The in vitro release study of allantoin, quercetin, and caffeic acid demonstrated the scaffolds’ potential for the simultaneous release of the active agents, making them promising candidates for non-invasive, ECM-mimetic, and multi-target wound therapy providing incremental improvements towards more efficient wound healing therapy.

## 2. Materials and Methods

### 2.1. Materials

The polymers from natural origin, gelatin from porcine skin (G, Type A, bioreagent) and sodium alginate (A, bioreagent), along with graphene oxide nanoparticles (GO, nanocolloids, dispperzion in water, n20/D1333), were all purchased from Sigma-Aldrich (St. Louis, MO, USA). The used crosslinking agent was 1-ethyl-3-(3-dimethylaminopropyl) carbodiimide hydrochloride (EDC, 98.0%), also obtained from Sigma-Aldrich. The active agents with high wound healing bioactivity, including allantoin, caffeic acid and quercetin as well as all reagents used to evaluate biocompatibility of the scaffolds via MTT assay on MRC5 cells and Caenorhabditis elegans (RPMI-1640 medium and supplements for cell proliferation) were sourced from Sigma-Aldrich, USA. For buffer preparation were used potassium hydrogen phosphates (Sigma-Aldrich, St. Louis, MO, USA), while all synthesis reactions were performed using ultra-distilled water.

### 2.2. Scaffolds Synthesis

In order to more closely mimic structure of native ECM, the scaffolds were engineered as natural-origin interpenetrating polymeric networks (IPNs) through a simple crosslinking reaction of gelatin by EDC in the presence of alginate. For the scaffolds (SCAFF) synthesis, 4% *w*/*v* solutions of gelatin and alginate were prepared separately in ultra-distilled water at 40 °C. To evaluate relationship between scaffolds composition and their properties, varying amounts of gelatin and alginate were used for the preparation of the initial polymeric mixture (Table 1). Into the initial polymeric mixture, 750 µL of a 1M EDC solution was added, stirred for 4 min at room temperature, and promptly poured into a Petri dish. After that the samples was stored at −18 °C for 24 h to complete the crosslinking reaction. Graphene oxide nanoparticles (GO) and the bioactive agents allantoin, quercetin, and caffeic acid (5% of the polymer content) were incorporated into the scaffolds by adding in the initial polymeric mixture. When the crosslinking reaction were completed, the samples were thoroughly washed with ultra-distilled water. The swollen scaffolds were then stored at −80 °C overnight and lyophilized. The chemical composition and abbreviated names of the scaffolds (SCAFF) are listed in Table 1.

### 2.3. Hydrogel Scaffolds Characterization

#### 2.3.1. Fourier Transform Infrared Spectroscopy (FTIR)

The chemical composition of the scaffolds was evaluated through FTIR spectroscopy, with FTIR spectra collected over a wavelength range of 700–4000 cm^−1^, using Thermo-Scientific Nicolet 6700 FTIR spectrometer, (Thermo Fisher Scientific, Uppsala, Sweden) equipped with a diamond crystal and ATR sampling method.

#### 2.3.2. Scanning Electron Microscopy (SEM)

Scanning Electron Microscope (SEM, Jeol JSM-7600 F, JEOL Ltd., Tokyo, Japan) was used to analyze the surface and cross-sectional morphology of the scaffolds. Before SEM imaging, the lyophilized scaffolds were coated with a layer of gold using BAL-TEC SCD 005 sputter coater (BAL-TEC AG, Balzers, Liechtenstein).

#### 2.3.3. Porosity Measurements

Porosity assessment of the scaffolds was performed using the solvent displacement method. The porosity was calculated using the true and bulk densities of the scaffolds, along with the density obtained by the Archimedes method, using glycerol (ρ = 1.2038 g/cm^3^) as the immersion fluid [20,21].

#### 2.3.4. Mechanical Testing

The mechanical properties of the scaffolds were evaluated through uniaxial tensile testing using a Galdabini Quasar 50, (Galdabini S.r.l., Cernusco sul Naviglio, Italy). The uniaxial tensile experiment was performed under compression with a 100 N load cell at room temperature. The Young’s modulus (E) of the scaffolds was determined from the linear part of the stress/strain curve. Each measurement was performed in triplicate, and the Young’s modulus was reported as the average value.

#### 2.3.5. In Vitro Swelling Study

The equilibrium degree of swelling of the scaffolds was assessed to examine their fluid absorption behavior capacity. To evaluate swelling capacity of the scaffolds, the freeze-dried scaffolds were immersed in phosphate buffer solution (pH of 7.4 at 37 °C) and weighed at specific intervals to determine their equilibrium degree, following a previously established equation [22,23,24].

#### 2.3.6. Water Contact Angle Measurements

The surface hydrophilicity of the scaffolds was examined using static water contact angle measurement via the sessile drop method, placing approximately 1 μL drop of MilliQ water on the surface of the scaffold. The measurements were conducted using a Theta Lite–Biolin Scientific Contact angle meter (Biolin Scientific, Stockholm, Sweden), offering a range from 0 to 180 degrees and accuracy +/− 0.1 degrees, +/− 0.01 mN/m with a 640 × 480 resolution camera and a maximum capture speed of 60 fps.

#### 2.3.7. Adhesiveness Test

The adhesive properties of the scaffolds were evaluated by attaching the swollen scaffold to the skin tissue of a moving joint, with the angle of flexion varying between 0° and 120° [25] Photographs documented the scaffold’s adhesion at different joint angles, including full extension at 0°, flexion at 45°, 90°, and 120°, as well as when adhered to the palm side of a finger.

#### 2.3.8. Biocompatibility Probes

##### In Vitro Cytotoxicity Assay

Cytotoxicity activities of the scaffolds were evaluated using the methods described previously [26]. In this procedure, 100 mg of the scaffolding material was sterilized, ground, and incubated in 10 mL of RPMI-1640 medium for 72 h at 37 °C. Then the obtained suspensions were centrifuged for 10 min at 5000 rpm using an Eppendorf Centrifuge 5804R (Eppendorf AG, Hamburg, Germany), and the supernatants were collected at various concentrations. The 3-(4,5-dimethylthiazol-2-yl)-2,5-diphenyltetrazoliumbromide (MTT) reduction assay was then performed using human lung fibroblasts MRC5 cell line obtained from ATCC. The cells were plated in a 96-well flat-bottom plate at a concentration of 1 × 104 cells per well grown in humidified atmosphere of 95% air and 5% CO_2_ at 37 °C and maintained as monolayer cultures in RPMI-1640 medium supplemented with 100 μg/mL streptomycin, 100 U/mL penicillin, and 10% (*v*/*v*) fetal bovine serum (FBS). After 24 h of incubation, media containing scaffold extracts at increasing concentrations (12.5%, 25%, 50%, and 100% (*v*/*v*)) were added to the cells. Control cells were maintained in 200 μL of growth medium without scaffold extracts. Following 48 h of incubation, cell cytotoxicity was assessed by the MTT reduction assay. The extent of MTT reduction to formazan within cells was measured by absorbance at 540 nm on a multiplate reader (Epoch 2000, BioTek, Winooski, VT, USA). The MTT assay was performed twice in quadruplicate, and the results were expressed as a percentage relative to the control (untreated cells), which was arbitrarily set to 100%. Finally, the cell viability percentage (%) of the scaffolds was calculated using the formula: (optical density of the treated group/optical density of the control group) × 100.

##### In Vivo Caenorhabditis Elegans Survival Assay

To study in vivo biocompatibility of the scaffolds *Caenorhabditis elegans* AU37 (glp-4; sek-1), obtained from the Caenorhabditis Genetics Center (CGC) at the University of Minnesota, Minneapolis, MN, USA was used. These nematodes were propagated under standard conditions on NGM media (Nematode Growth Medium) with an *Escherichia coli* OP50 bacterial suspension, as described earlier [27]. For the experiments, small Petri dishes were used. In each petri dish, 4 mL of NGM medium was added, followed by 60 μL of an overnight culture of *E. coli* OP50, which was spread and incubated for 24 h at 37 °C. The tested materials were cut into thin slices and submerged in M9 medium for an hour before being placed on the bacterial lawn 20 to 30 L4 larvae *C. elegans* were then added to the NGM medium with *E. coli* and the tested materials and incubated for 4 days at 25 °C. Every 24 h, the worms were examined under a microscope to count the number of dead and alive worms. As a negative control, only an NGM plate with *E. coli* and worms was used.

To determine whether the scaffolding materials affect the growth rate of *C. elegans*, a new set of experiments was conducted. L4 larvae worms were cultivated under the same conditions as in the previous experiment. After 24 h, the worms were measured using SenseCell Olympus program (1.17). The negative control consisted of *C. elegans* with only *E. coli*. The results are presented as a percentage of body length relative to this control.

In both experiments, each material was tested in triplicate, and the experiments were repeated twice.

#### 2.3.9. In Vitro Simultaneous Controlled Release Study

The release profiles of allantoin, caffeic acid, and quercetin from the scaffolds were analyzed using a UV/Vis spectrophotometer (Shimadzu UV-1800, Kyoto, Japan). The scaffolds loaded with these active agents were placed in a basket stirrer containing 800 mL of release medium (phosphate buffer, pH 7.4 at 33.5 °C) simulating physiological conditions. The concentrations of the released allantoin, quercetin, and caffeic acid were tracked by recording the absorbance of the release medium at specific time intervals, with the maximum absorption wavelengths (λ_max_) values set at 204 nm for allantoin, 380 nm for quercetin, and 280 nm for caffeic acid [28,29,30].

## 3. Results and Discussion

### 3.1. Preparation of the Scaffolds

**Scheme 1:**
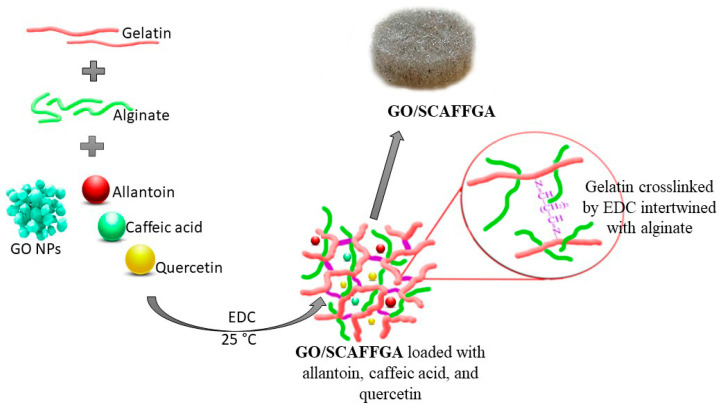
Synthesis route of GO/gelatin/alginate scaffolds loaded with the active agents—allantoin, quercetin and caffeic acid (details: photographs of the scaffold).

The concept of ECM-mimetic and specific wound healing phase-target therapy based on natural-origin scaffolds and the multiple bioactive compounds with high wound healing potential is presented. This therapeutic concept offers both structural and biophysiological support through all specific phases of the skin tissue regenerative process in a single approach. Guided by the inspiration of the unique properties of the native ECM structure, the porous scaffolds were engineered as interpenetrating polymeric networks (IPNs) based on natural polymers—alginate and gelatin. The zero-length crosslinking method was used for the synthesis of the scaffolds, where EDC was used as crosslinker for gelatin due to its ability to form amide bonds between gelatin chains resulting in formation of stable crosslinks within gelatin. The interpenetrating polymeric networks (IPNs) were constructed by gelatin crosslinked with EDC, intertwined with linear chains of alginate, and reinforced with GO nanoparticles, as illustrated in Scheme 1. The scaffolds engineered through IPN formation are recognized as promising forms for tissue engineering applications because they simultaneously stimulate cellular processes through replicated ECM structure, and provide enough free space into the polymeric network for loading and release of the bioactive molecules with high potential to improve tissue regenerative process. The GO/gelatin/alginate scaffolds loaded with allantoin, caffeic acid and quercetin were engineered as multi-bioactive IPNs to provide both ECM-mimetic and specific wound healing phase-target therapy in a single entity.

In order to evaluate and optimize the chemical, mechanical, morphological, biological, swelling and drug release properties of the scaffolds for potential wound healing application, varying amounts of gelatin and alginate were used in their preparation. To obtain biocompatible scaffolds for wound healing applications pure gelatin was selected due to its high bioactivity, originating from RGD sequences that promote essential cellular processes, including adhesion, migration and proliferation. Pure gelatin was also selected as a control for evaluating the impact of reduced gelatin content on the scaffold’s properties and functionality. The scaffold composition with equal amounts of natural polymers—alginate and gelatin was formulated to achieve a balance between flexibility of gelatin and stiffness of alginate. This equal alginate/gelatin ratio was selected to evaluate the synergistic effect of both polymers, not only on mechanical properties but also on overall functionality of the scaffold, including biocompatibility, morphology, porosity and swelling capacity. Additionally, the scaffold composition with a higher content of alginate was selected to evaluate how a reduced content of gelatin influences the bioactivity of the scaffold providing insights into whether the decreased content of RGD sequences, could be compensated by improvements in structural, morphological properties, porosity and swelling capacity of the scaffolds. The chemical composition and abbreviated names of the scaffolds (SCAFF) are listed in Table 1.

### 3.2. Structural Characteristics of the Scaffolds—FTIR Analysis

**Figure 1:**
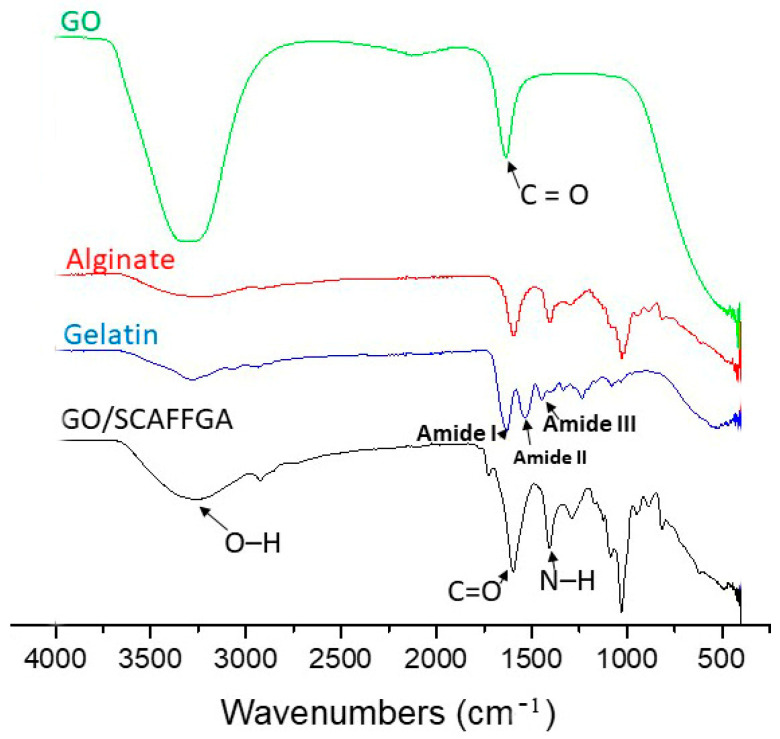
FTIR spectra of pure GO, alginate, gelatin and the scaffold based on gelatin and alginate, reinforced with GO nanoparticles (GO/SCAFFGA).

FTIR spectroscopy was used to identify the chemical composition and molecular interactions within gelatin/alginate scaffolds reinforced with GO nanoparticles. The FTIR spectra of the pure scaffolds’ components, including GO, gelatin, and alginate, and of the synthesized scaffold based on gelatin crosslinked with EDC, intertwined with alginate chains and reinforced with GO nanoparticles (GO/SCAFFGA) are presented in Figure 1. The FTIR spectrum of GO/SCAFFGA displays characteristic IR absorption bands for each component of the scaffolds’ composition—gelatin, alginate and GO. Thus, the presence of gelatin into the scaffolds is confirmed by the IR peaks at 1629 cm^−1^, 1510 cm^−1^, and 1320 cm^−1^ corresponding to the C=O stretching vibrations of the peptide bonds in gelatin (Amide I), N–H bending vibration combined with C–N stretching vibrations (Amide II), and C–N stretching coupled with N–H bending vibrations (Amide III), as well as by IR signal at around 3290 cm^−1^ attributed to the O–H groups present in the gelatin structure [31,32]. Additionally, FTIR spectrum of GO/SCAFFGA also reveals increased intensity of the Amide I, Amide II, and Amide III peaks in GO/SCAFFGA compared with the pure gelatin, indicating increased number of amide bonds formed by crosslinking of gelatin with EDC. The FTIR spectrum of GO/SCAFFGA scaffold also features specific bands representing alginate including signals at 3250 cm^−1^ (O–H stretching), 1300 cm^−1^ (C–O stretching), 1100 cm^−1^ (C–C stretching), and 1030 cm^−1^ (C–O–C stretching) [33,34]. The shifts to the lower wavenumber of the IR absorption bands related to the stretching vibration of the N–H group bonded to the O–H group indicate intermolecular interactions and good chemical compatibility between gelatin and alginate [35,36,37]. The FTIR spectrum of pure GO reveals the presence of oxygen-containing groups by signals at 3340 cm^−1^ which is attributed to the stretching vibrations of the OH groups, while the absorption peak at 1730 cm^−1^ can be attributed to the C = O stretching of the carbonyl groups in GO nanoparticles [38,39]. The observed IR absorption bands and spectral changes in FTIR spectrum of GO/SCAFFGA scaffold indicate successful crosslinking of gelatin with EDC through increased intensity of the Amide I, Amide II, and Amide III peaks compared with pure gelatin, physical crosslinking through hydrogen bonding between the hydroxyl groups of alginate and the carboxyl groups of gelatin, as well as successful incorporation of GO nanoparticles into the scaffolds through increased intensity of the carbonyl groups peak at 1730 cm^−1^ in GO/SCAFFGA spectrum.

### 3.3. Morphology of the Scaffolds—SEM Analysis

**Figure 2:**
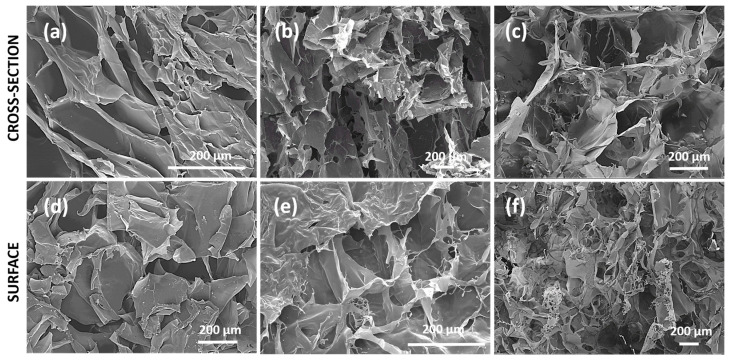
SEM micrographs of cross-section of (**a**) GO/SCAFFG, (**b**) GO/SCAFFGA and (**c**) GO/SCAFFG3A and of surface of (**d**) GO/SCAFFG, (**e**) GO/SCAFFGA and (**f**) GO/SCAFFG3A scaffold.

The morphology of the scaffolds is a crucial property for their application in tissue regeneration/engineering due to it influences not only cellular processes and bio-interactions but also mechanical stability and ability to loading and release of the bioactive agents. The cross-sectional and surface morphology of gelatin/alginate scaffolds reinforced with GO nanoparticles were examined by SEM and obtained micrographs are presented in Figure 2. The SEM micrographs of the scaffolds reveal porous morphology with variable form and interconnected pores which are suitable for tissue regeneration application. The scaffold based on gelatin reinforced with GO nanoparticles (Figure 2a) reveals lamellar morphology close to the lamellar structure of the native tissue, favorable for cellular processes, and interactions as well as for loading and release of the bioactive agents. Incorporation of alginate into the scaffold composition modifies porosity and pattern of cross-sectional and surface morphology. The scaffolds with alginate in their composition possess larger, irregularly shaped pores. The formation of the larger pores by adding alginate into the scaffold composition can be attributed to the reduced content of chemically crosslinked gelatin with EDC as well as to hydrophilic nature of alginate, resulting in forming large ice crystals in the freeze-drying method [40]. Obtained SEM results indicate design of the scaffolds with different morphologies by simple varying their chemical composition in order to meet the requirements of their final application.

### 3.4. Porosity of the Scaffolds

Porosity directly influences the biological and mechanical properties of the scaffolding biomaterial, affecting its overall functionality for tissue regeneration [41]. For skin or soft tissue regeneration, porosity around 90% offers sufficient mechanical support and favorable environment for successful cell populations and tissue growth throughout the scaffold. The porosity values obtained for the gelatin/alginate scaffolds reinforced with GO nanoparticles are presented in Table 2 indicating their dependence on the chemical composition of the scaffolds. Based on the results, it is evident that introducing of alginate into the scaffolds composition increases their porosity probably due to a reduced degree of crosslinking caused by the decreased content of chemically crosslinked gelatin as well as due to hydrophilic nature of alginate. The porosity values of the gelatin/alginate scaffolds reinforced with GO nanoparticles, ranging between 93% and 96%, are favorable for soft tissue regeneration including skin tissue regeneration and wound healing applications.

### 3.5. Mechanical Properties of the Scaffolds

The mechanical properties of the scaffolding biomaterials play a crucial role in determining their biological potential for cellular processes and tissue regeneration [42,43,44] Mechanical properties of the scaffolds must be precisely engineered to match the characteristics of the target tissue to optimize cellular processes, tissue integration and overall healing outcomes. For wound healing application, scaffolds need to be soft enough to mimic the ECM of skin and soft tissue. Various studies have demonstrated that the biomechanical properties of skin tissue are influenced by factors such as age, anatomical location, and the measurement technique employed. [45,46,47]. Literature research data indicate that the Young’s modulus of skin ranges from 10 kPa to 50 MPa, suggesting that scaffolds for wound healing should exhibit mechanical properties within this spectrum [48]. Obtained results of uniaxial tension experiments for the scaffolds, listed in Table 2, revealed Young’s modulus (E) values ranging between 1.10 and 2.90 MPa. These results demonstrate relationship between the scaffolds composition and their biomechanical properties, highlighting the impact of the crosslinking degree. The highest value of Young’s modulus was observed for the scaffold based on gelatin reinforced with GO nanoparticles as result of the formation of chemical crosslinks between gelatin and EDC. The incorporation of alginate into the scaffold composition decreases values of Young’s modulus probably due to reduced amount of chemically crosslinked gelatin. Obtained values of Young’s modulus of the gelatin/alginate scaffolds reinforced with GO nanoparticles are comparable to those of normal human skin suggesting mechanical suitability of the scaffolds for wound healing applications.

### 3.6. Swelling Properties of the Scaffolds

The capacity of scaffolds to absorb physiological fluids is a crucial factor which influences effectiveness of healing process and should be precisely engineered to effectively manage exudate levels, enhancing the overall wound healing treatment. The ideal scaffold for wound healing should effectively absorb wound exudates and simultaneously provide a moist wound environment suitable for healing process [49]. Excess exudate can cause maceration of surrounding skin tissue and delaying healing while lower moisture can lead to wound desiccation. Thus, the swelling capacity of the scaffolds was evaluated in vitro in a buffer solution with a pH of 7.40. Obtained results are presented in Table 2 as values of equilibrium degree of swelling (*q_e_*) indicating their dependence on the scaffold composition. The obtained *q_e_* values are in the range between 11 and 23, highlighting that the introduction of alginate into the scaffolds’ composition increases their swelling capacity. The scaffold with predominant content of alginate provide absorption of over 20 times its initial weight in the buffer solution due to the lower content of chemically crosslinked gelatin by EDC and the high hydrophilicity of alginate. Additionally, both gelatin and alginate are rich with hydrophilic groups in their structure such as -OH and -NH_2_ groups, which contribute to the fluid absorption capacity confirming these scaffolds suitable for wound healing applications.

### 3.7. Hydrophilicity of the Scaffolds

Hydrophilicity of the scaffolds is essential property for efficient tissue regeneration due to influences cellular processes including adhesion, migration and proliferation as well as protein adsorption which are important phases in tissue regeneration process. Hydrophilicity of the scaffolds surface was evaluated by contact angle measurements, with results recorded at 0 s. The obtained values of the contact angle presented in Table 2. are less than 90° revealing fully hydrophilic surface of the gelatin/alginate scaffolds reinforced with GO nanoparticles. The fully hydrophilic nature of the scaffold surface, resulting from porous structure and the presence of hydrophilic functional groups from gelatin and alginate, makes the scaffolds highly favorable for skin tissue regeneration.

### 3.8. Adhesion Properties to the Skin Tissue of the Scaffolds

**Figure 3:**
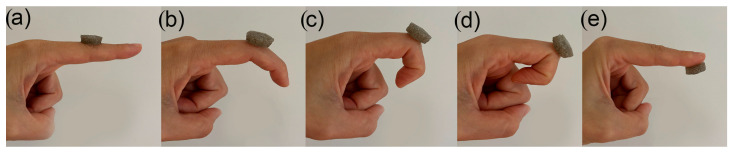
Photographs of scaffolds based on gelatin and alginate reinforced with GO nanoparticles attached on (**a**) finger extended at 0 °, (**b**) finger flexed to 45 °, (**c**) finger flexed to 90 °, (**d**) finger flexed to 120 °, (**e**) palm side of finger.

The tissue adhesive properties of scaffolds for tissue engineering/regeneration provide multiple advantages for their application in wound healing, including better adaptability to curved, uneven or folded surface of complex wounds, ability to protect wound from bacteria and other pathogens as well as from external environment exposure, applicability without sutures, glue or staples that can cause secondary damages of tissue [50]. The adhesive properties of the scaffolds were evaluated by their attaching to skin tissue on a moving joint. Obtained photographs are presented in Figure 3. indicating stable attachment of the scaffolds to skin tissue at intersection angles ranging from 0° to 120° as well as at reverse position. Adhesion of a scaffold requires interaction between its surface and the recipient tissue which can be achieved by molecular interactions (such as ionic, covalent, hydrogen, Van der Waals and hydrophobic bonding) and chain penetration and entanglement [51,52,53]. The hydrogen bond is the driving force of supramolecular adhesives and can be used as a supplemental force for scaffolds based on protein or polysaccharide [54]. Thus, gelatin and alginate provide stable adhesion to the skin tissue due to presence of numerous hydrogen bond-forming sites, including hydroxyl, carboxyl, and amine groups. The scaffolds based on gelatin/alginate reinforced with GO nanoparticles reveal spontaneous, stable adhesion to skin tissue probably due to both hydrogen bonding and electrostatic interactions between the amino groups from gelatin and the oppositely charged phospholipids in skin tissue membranes [55].

### 3.9. Biocompatibility Assays of the Scaffolds

**Figure 4:**
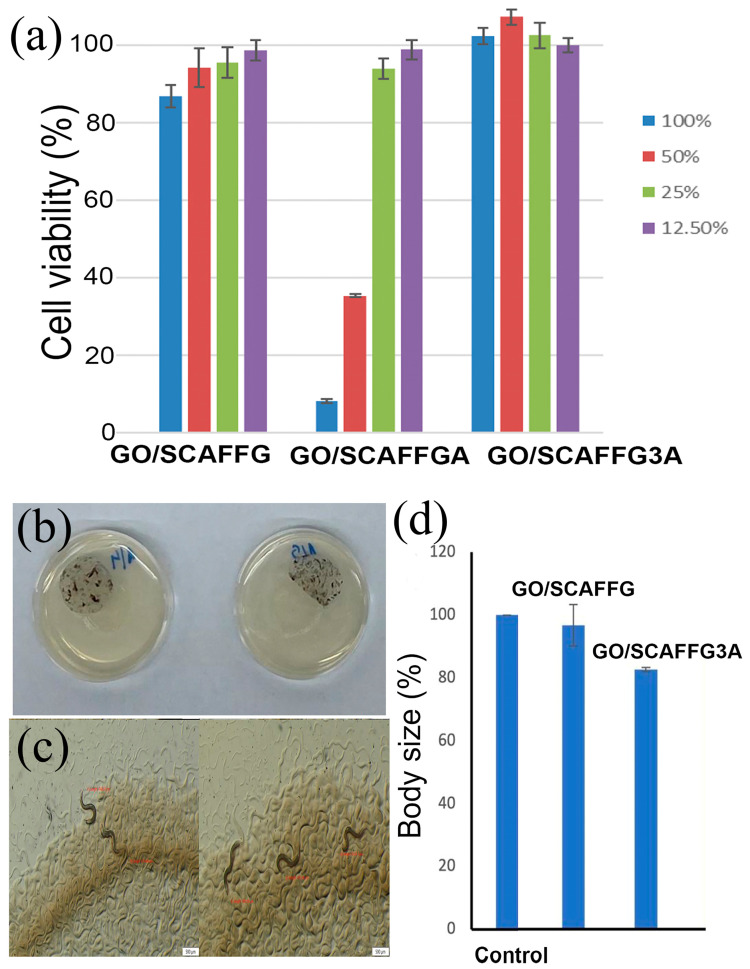
(**a**) MRC5 cell viability in the presence of 12.50%, 25%, 50%, and 100% *v*/*v* of extracts of gelatin/alginate scaffolds reinforced with GO nanoparticles, (**b**) Photographs of plates with *E. coli*, gelatin/alginate scaffolds reinforced with GO nanoparticles after *C. elegans* were added, (**c**) Measurement of worms after 24 h using SenseCell Olympus program, (**d**) Percentage of body length of worms relative to control.

Biocompatibility is one of the mandatory requirements in selecting scaffolds for tissue regeneration/engineering including wound healing application. The concept of biocompatibility includes not only bio-inertia but also biofunctionality and biostability [56]. In this regard, the biocompatibility of the scaffolds was evaluated by both in vitro and in vivo studies. The in vitro evaluation of scaffolds cytocompatibility was performed using the MTT test on the MRC5 cell line, while the in vivo evaluation of biocompatibility was assessed using the *C. elegans* survival assay. The results are presented in Figure 4.

For the in vitro evaluation, different concentrations of the scaffold extracts were applied to MRC5 cells. The results of cell viability (Figure 4a) indicated that all extracts from GO/SCAFFG and GO/SCAFFG3A scaffolds, as well as the 25% and 12.5% extracts of GO/SCAFFGA are within a biologically acceptable range for cell viability, confirming the in vitro cytocompatibility of the scaffolds. The 100% and 50% extracts of GO/SCAFFGA scaffold demonstrated the significantly lower cell viability probably due to its slightly reduced swelling capacity and porosity compared to the GO/SCAFFG3A scaffold which is insufficient to compensate the decreased bioactivity resulting from the reduced content of RGD sequences compared to the GO/SCAFFG scaffold. The scaffold with the predominant content of alginate beyond good cytocompatibility showed the proliferative effect on MRC5 cells which is of great importance for tissue regenerative process. The obtained results of MRC5 cell viability indicate that the scaffold based on gelatin (GO/SCAFFG) as well as scaffold with predominant content of alginate (GO/SCAFFG3A) are optimal and safe for biomedical applications and they were used in additional in vivo biological evaluation by *C. elegans* survival assay.

The nematode *C. elegans* was selected for in vivo testing of the scaffolds’ biomedical potential due to its benefits over mammalian models in predictive toxicology and drug discovery, such as fewer regulatory requirements, short life cycle with high reproduction rate, cost-effective cultivation, notable genetic and tissue similarities to humans as well as rapid response to treatments due to its simple body structure and fast metabolism [57,58]. To perform in vivo testing, the sliced scaffolds were incubated with *C. elegans* larvae for 4 days (Figure 4b,c). Every 24 h, the worms were analyzed under a microscope to determine the number of dead and alive worms. No mortality was detected among the worms throughout the incubation period, indicating that gelatin/alginate scaffolds reinforced with GO nanoparticles were not toxic to *C. elegans* under these conditions. The worms spent time both under the scaffolds and on other parts of the plates, indicating that the scaffolds did not affect the *C. elegans* life cycle.

In order to determine whether the scaffolds affect the growth rate of *C. elegans*, the body length of worms cultivated 24 h in the presence of the scaffolds was measured. The negative control was *C. elegans* with only *E. coli*. The typical body length of adult hermaphrodite *C. elegans* is 1250–1400 μm. The control worms had an average length of 1263.95 μm. Worms exposed to the scaffolds exhibited body lengths reduced by 20% compared to the control (Figure 4d). The obtained results of both in vitro and in vivo biological evaluations confirm good biocompatibility of the gelatin/alginate scaffolds reinforced with GO nanoparticles. 

### 3.10. In Vitro Simultaneous Release Properties of the Scaffolds

**Figure 5:**
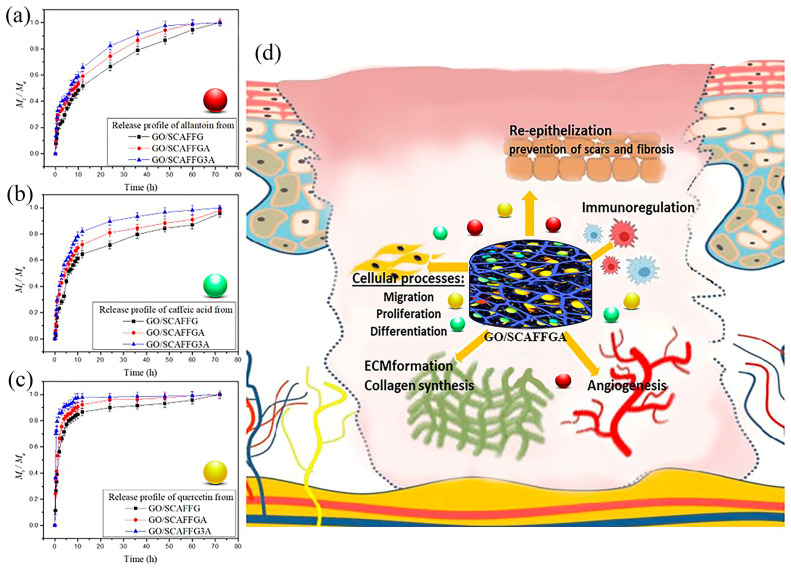
The release profiles of (**a**) allantoin, (**b**) caffeic acid, and (**c**) quercetin from the scaffolds based on gelatin and alginate reinforced with GO nanoparticles, (**d**) illustration of specific wound healing phase-target including inflammation, proliferation and tissue remodeling by GO/SCAFFGA.

The core idea of the proposed scaffolds design is providing regeneration of complete skin tissue structures by bio-activation across all phases of wound healing process using a single system. The scaffolds designed by this strategy offer both passive and active support during the wound healing process. The 3D highly porous ECM-mimetic structure of the scaffolds provides bio-structural support for health tissue formation and integration in three dimensions, while stimulation of all specific wound healing phases was achieved by simultaneous release of the active agents including allantoin that stimulates local granulation, inflammatory response, collagen and ECM synthesis, caffeic acid that improves re-epithelization, inhibits peroxidation of lipides, provides myeloperoxidase activity and quercetin which prevents scars and fibrosis (Figure 5d). This therapeutic strategy provides stimulation of all specific wound healing phases including inflammation, proliferation and tissue remodeling through a single approach (Figure 5d). To evaluate potential of the scaffolds for simultaneous release of the active agents, the release study of allantoin, caffeic acid, and quercetin from the scaffolds was conducted in vitro in simulated physiological conditions at pH 7.4 and 33.5 °C. The release data, presented in Figure 5a–c, and Table 3 and Table 4, demonstrate the significant impact of both composition of the scaffold and the type of the active agent on the release profiles.

As can be seen from Figure 5. rapid release of the active agents from the scaffolds was detected within the first 6 h of the release. Thus, 80% of quercetin, 52% of caffeic acid, and 38% of allantoin were released from the scaffold based on gelatin reinforced with GO nanoparticles, while the scaffold with predominant content of alginate released 92% of quercetin, 62% of caffeic acid and 46% of allantoin during this initial release period. The observed release phenomenon indicates that the introduction of alginate into the composition of the scaffold results in a faster release of the active agents. This can be attributed to the hydrophilic nature of natural polymer alginate and its great swelling capacity that accelerate the diffusion and release rate of active molecules through swollen polymeric network.

The initial rapid release phase was followed by a slower release of the active agents from all scaffolds, maintaining the same release pattern. It was also observed that the type of the active agent determined the release profile. Thus, the highest release rate was obtained for quercetin (Figure 5c), moderate release rate for caffeic acid (Figure 5b), and the slowest release for allantoin (Figure 5a). These differences in the obtained release profiles can be attributed to the distinct chemical and physical properties of the active agents, as well as the presence of the specific functional groups. The investigated gelatin/alginate scaffolds reinforced with GO nanoparticles and loaded with multiple active agents demonstrated potential for simultaneous release of allantoin, quercetin, and caffeic acid over 72 h, highlighting that the release profile can be optimized by adjusting the chemical composition of the scaffold. In this way, improved wound healing treatment could be achieved through the application of a single system that offers both ECM-mimetic support and simultaneous release of the active agents with the high wound healing potential.

## 4. Conclusions

This study presented the innovative wound healing strategy by engineered graphene oxide/gelatin/alginate scaffolds loaded with allantoin, quercetin and caffeic acid. The synergistic activity of 3D porous polymeric scaffolds and multiple active agents suggests behavior closer to the natural microenvironment and both ECM-mimic and specific wound healing phase-target therapy in a single approach. The natural origin polymeric scaffolds revealed highly porous interconnected microstructure, hydrophilic nature, both in vitro and in vivo biocompatibility leading to the stimulation of cellular processes and health tissue formation and integration in three dimensions through replicated ECM-structure of native skin tissue. The scaffolds showed simultaneous release of the active agents providing bio-stimulation of each phase in wound healing process, from inflammation and proliferation to the skin tissue remodeling. Our findings highlight the potential of the innovative, non-invasive wound healing strategy to improve healing outcomes and provide incremental improvements towards more efficient wound healing therapy.

## Figures and Tables

**Table 1:** The chemical composition and abbreviated names of the scaffolds (SCAFF).

Sample	Gelatin Solution (4% *w*/*v*) (mL)	Sodium Alginate Solution (4% *w*/*v*) (mL)
GO/SCAFFG	30	0
GO/SCAFFGA	15	15
GO/SCAFFG3A	7.5	22.5

**Table 2:** The values of the porosity (%), Young’s Modulus (MPa), Contact angle and Equilibrium degree of swlling (*q_e_*) of the scaffolds.

Scaffold	Porosity (%)	Young’s Modulus (MPa)	Contact Angle (°)	Equilibrium Degree of Swelling (*q_e_*)
GO/SCAFFG	93.18 ± 4.62	2.90 ± 0.15	76.52 ± 2.91	11.04 ± 0.56
GO/SCAFFGA	94.97 ± 4.67	2.47 ± 0.11	0.00 ± 3.29	18.50 ± 0.62
GO/SCAFFG3A	96.09 ± 4.72	1.10 ± 0.05	0.00 ± 4.21	23.44 ± 0.68

**Table 3:** The percentage of the released active agents from the scaffolds after 6 h of release.

Scaffold	Released Quercetin (%)	Released Caffeic Acid (%)	Released Allantoin (%)
GO/SCAFFG	79.98 ± 3.92	51.82 ± 2.62	37.92 ± 1.88
GO/SCAFFGA	85.09 ± 4.23	57.22 ± 2.82	45.45 ± 2.25
GO/SCAFFG3A	91.88 ± 4.56	62.22 ± 3.11	45.95 ± 2.28

**Table 4:** The release data for initial and slow stage of the active agents release from the scaffolds.

**Scaffold**	**Initial Stage of Release**					
	**Quercetin**		**Caffeic Acid**		**Allantoin**	
	**mg/6 h**	**mg/h**	**mg/6 h**	**mg/h**	**mg/6 h**	**mg/h**
GO/SCAFFG	47.99 ± 2.36	7.99 ± 0.40	31.09 ± 1.55	5.18 ± 0.25	22.75 ± 1.10	3.79 ± 0.18
GO/SCAFFGA	51.05 ± 2.55	8.51 ± 0.42	34.33 ± 1.71	5.72 ± 0.38	27.27 ± 1.35	4.54 ± 0.23
GO/SCAFFG3A	55.12 ± 2.77	9.19 ± 0.45	37.33 ± 1.84	6.22 ± 0.34	27.57 ± 1.37	4.59 ± 0.23
	**Slow Stage of Release**					
	mg/66 h	mg/h	mg/66 h	mg/h	mg/66 h	mg/h
GO/SCAFFG	12.01 ± 0.60	0.18 ± 0.01	28.91 ± 1.41	0.44 ± 0.02	37.25 ± 1.85	0.56 ± 0.03
GO/SCAFFGA	8.95 ± 0.45	0.14 ± 0.01	25.67 ± 1.26	0.39 ± 0.02	32.73 ± 0.64	0.50 ± 0.02
GO/SCAFFG3A	4.88 ± 0.25	0.07 ± 0.01	22.67 ± 0.10	0.34 ± 0.01	32.43 ± 0.45	0.49 ± 0.01

## Data Availability

Data are contained within the article.
